# Worsening glycemic control in youth with type 2 diabetes during COVID-19

**DOI:** 10.3389/fcdhc.2022.968113

**Published:** 2022-09-09

**Authors:** Sonum Bharill, Tyger Lin, Alexander Arking, Elizabeth A. Brown, Margaret West, Kelly Busin, Sheela N. Magge, Risa M. Wolf

**Affiliations:** Department of Pediatrics, Division of Endocrinology, Johns Hopkins University School of Medicine, Baltimore, MD, United States

**Keywords:** type 2 diabetes (T2D), Coronavirus – COVID-19, A1C (or HbA1c), hemoglobin A1c, glycemic control, adolescent

## Abstract

**Introduction:**

The COVID-19 pandemic has disproportionately affected minority and lower socioeconomic populations, who also have higher rates of type 2 diabetes (T2D). The impact of virtual school, decreased activity level, and worsening food insecurity on pediatric T2D is unknown. The goal of this study was to evaluate weight trends and glycemic control in youth with existing T2D during the COVID-19 pandemic.

**Methods:**

A retrospective study of youth <21 years of age diagnosed with T2D prior to March 11, 2020 was conducted at an academic pediatric diabetes center to compare glycemic control, weight, and BMI in the year prior to the COVID-19 pandemic (March 2019-2020) to during COVID-19 (March 2020-2021). Paired t-tests and linear mixed effects models were used to analyze changes during this period.

**Results:**

A total of 63 youth with T2D were included (median age 15.0 (IQR 14-16) years, 59% female, 74.6% black, 14.3% Hispanic, 77.8% with Medicaid insurance). Median duration of diabetes was 0.8 (IQR 0.2-2.0) years. There was no difference in weight or BMI from the pre-COVID-19 period compared to during COVID-19 (Weight: 101.5 v 102.9 kg, p=0.18; BMI: 36.0 v 36.1 kg/m2, p=0.72). Hemoglobin A1c significantly increased during COVID-19 (7.6% vs 8.6%, p=0.0002)

**Conclusion:**

While hemoglobin A1c increased significantly in youth with T2D during the COVID-19 pandemic, there was no significant change in weight or BMI possibly due to glucosuria associated with hyperglycemia. Youth with T2D are at high risk for diabetes complications, and the worsening glycemic control in this population highlights the need to prioritize close follow-up and disease management to prevent further metabolic decompensation.

## Introduction

The COVID-19 pandemic resulted in a worldwide lockdown, during which time most children and adolescents attended school virtually (1). Rates of overweight and obesity among children and adolescents during this time have increased significantly worldwide, owing to an increase in higher calorie processed foods, decreased activity level, increasing food insecurity and increased rates of stress and anxiety due to the pandemic (2, 3). Moreover, the COVID-19 pandemic has disproportionately affected minority and lower socioeconomic status populations (4), who have higher rates of obesity, type 2 diabetes (T2D), and associated complications (5, 6).

The obesity epidemic over the last 20 years has led to an increase in youth onset T2D, most notably in non-Hispanic black, Asian/Pacific Islanders and American Indians (5–9). Almost half of new-onset diabetes presenting in adolescents is attributed to type 2 diabetes (10, 11), and youth onset type 2 diabetes is often characterized by a more rapidly progressive decline in B-cell function compared to adults (12). Studies have also demonstrated a high rate of diabetes-related complications in the majority of youth onset type 2 diabetes that is present by young adulthood (13). Management of pediatric T2D is aided by adherence to schedules and parental support (14), and negatively affected by depression, sedentary behavior, and a diet rich in processed foods (15). These are all factors which have been severely disrupted during the COVID-19 pandemic.

The impact of virtual school, decreased activity level, and worsening food insecurity on pediatric T2D has not been thoroughly analyzed, but there is concern for worsening pediatric obesity, thereby exacerbating T2D and worsening glycemic control. A recent study demonstrated an increased incidence of diabetic ketoacidosis (DKA) at initial T2D presentation during the COVID-19 pandemic (16), however there is little data regarding changes in glycemic control among adolescents with known T2D during the COVID-19 pandemic. Among youth with known type 1 diabetes (T1D), studies have shown an increase in DKA with maintenance of overall glycemic control during the COVID-19 pandemic (17, 18). Clinical experience during the COVID-19 pandemic suggested an increase in overweight and obesity with worsening glycemic control in patients with T2D diagnosed prior to the COVID-19 pandemic. This study sought to evaluate weight trends and glycemic control in youth with known T2D during the COVID-19 pandemic compared to the prior year.

## Materials and methods

### Participants

A retrospective chart review of youth with T2D seen at an urban academic pediatric diabetes center was conducted to compare glycemic control in the year prior to and the first year of the COVID-19 pandemic. Patients younger than 21 years old, diagnosed with T2D anytime prior to March 11, 2020 and had an endocrine visit in the pre-COVID-19 period with confirmed negative diabetes-associated antibodies (glutamic acid decarboxylase-65, insulin auto-antibody, islet antigen-2 antibody, and/or islet cell antibody) were included in the study. Patients with steroid- or medication-induced diabetes, prediabetes, and post-transplant diabetes were excluded. We compared parameters from visit(s) during the year prior to the COVID-19 pandemic (from March 11, 2019 to March 10, 2020) to those from visit(s) during the COVID-19 period (March 11, 2020 to March 10, 2021). Participants served as their own historical controls. Because this analysis focuses on the changes from the pre-COVID-19 to the COVID-19 period, patients with no follow up in the COVID-19 period were excluded from the analysis (n=3). Per the American Diabetes Association guidelines for 2019 and 2020, the goal HbA1c was <7.0%. This study was approved by the institutional review board at the Johns Hopkins Hospital in adherence to the Declaration of Helsinki, with a waiver of consent.

### Data source and variables

Clinical diabetes-related variables were manually extracted from the electronic medical record (EMR). Age at diagnosis, duration of diabetes, weight, body mass index (BMI), and hemoglobin A1c (HbA1c) values from endocrine clinic visits were recorded, as well as modality (in-person or virtual) of visit. In the COVID-19 period when visits may have been *via* telemedicine and weight, BMI, or HbA1c measurements may not have been available from an endocrine clinic visit, these measures were taken from a non-endocrine visit in proximity to the endocrine visit. Hospital admissions and emergency department (ED) visits per patient during the pre-COVID and COVID periods were tallied from the EMR and Care Everywhere, allowing access to medical records from outside of the primary institution. Where available, we gathered data on virtual or in-person school, and whether parents were at home with the child.

### Statistical analysis

Variables were assessed for normal distribution using the Shapiro-Wilk test. Descriptive statistics were summarized using mean and the standard deviation for continuous normal variables, median and interquartile range for non-normally distributed, and percentage for categorical variables. The Wilcoxon signed rank test was used to compare the number of ED, hospital, endocrine, and missed visits in the two periods. A paired t-test was used to compare the mean weight, BMI, BMI Z-score and HbA1c in the two periods for participants with follow up in the pre and COVID periods. The 3 patients that did not have follow up measures in the COVID-period and were thus excluded, did not differ significantly at baseline in a two-sample *T* test from those who did have follow up in HbA1c, BMI, BMI z-score or weight. Regression analysis was conducted using a multivariable mixed effects model with a randomly varying intercept. Changes in in trajectory of weight, BMI, BMI Z-score, and HbA1c were assessed during the pre-COVID-19 year to the year with COVID-19 restrictions using a linear spline model with a knot at March 11, 2020. Descriptive variables including gender, age, race, ethnicity, duration of diagnosis, insurance type, if a parent was home in the COVID-19 period, and if the subject was in virtual school were tested for significance in the models as covariates and for their interaction with the slope before and after the knot. SAS version 15.2 was used in all analysis.

## Results

### Participants and baseline clinical characteristics

At total of 63 youth with T2D diagnosed prior to the start of the COVID-19 “stay-at-home” order on March 11^th^, 2020 were included in this analysis. As shown in **Table 1**, median age of study participants was 15.0 (IQR 14-16) years, 59% were female, 74.6% were African American, and 77.8% had public insurance. At the start of the study time frame, the median duration of diabetes was 0.8 (IQR 0.2-2.0) years, and participants’ median baseline HbA1c was 7.2 (IQR 6.1-9.6) %. Baseline mean weight was 102.7 ± 30.5 kilograms and median baseline BMI was 35.0 (IQR 29.7-43.8) kg/m^2^. During the pre-COVID year, there were on average 2.2 measures for each of the variables (weight, BMI, BMI z-score, HbA1c), with a range of 1-4 measures per participant. During the COVID year, there were on average 1.4 with a range of 0-4 measures per participant for weight, BMI, BMI z-score, and a range of 0-3 measurements for HbA1c. Pre-COVID, the median number of endocrine visits attended was 2, with a median of one missed endocrine visit. Median number of hospital admissions in the pre-COVID period among study participants was 0 (range 0-6), and average number of ED visits was 0 (range 0-6). In unadjusted analysis, a lower baseline BMI Z-score was significantly associated with female sex (p=0.011) and longer duration of diabetes diagnosis (p=0.0007).

**Table 1:** Baseline demographic and clinical characteristics (n=63).

Variable	Value
Age in years, median (Q1-Q3)	15.0 (14–16)
Diagnosis duration in years, median (Q1-Q3)	0.8 (0.2-2.0)
Age in years at diagnosis, median (Q1-Q3)	14 (12–15)
**Gender, n (%)**	
Female	37 (59%)
**Race and ethnicity, n (%)**	
Non-Hispanic Black	47 (74.6%)
Non-Hispanic White	6 (9.5%)
Hispanic	9 (14.3%)
Other	1 (1.6%)
**Insurance, n (%)**	
Private	14 (22.2%)
Public	49 (77.8%)
Weight in Kg, mean (SD)	102.7 (30.5)
BMI, median (IQR)	35 (29.7-43.8)
BMI Z-score, median (IQR)	2.3 (1.9-2.7)
Hemoglobin A1c %, median (IQR)	7.2 (6.1-9.6)

### Changes in clinical characteristics during COVID-19

**Figure 1:**
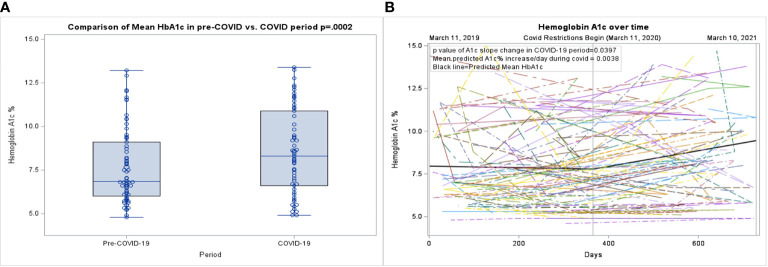
**(A)** Box plot of the pre-COVID-19 period compared to the COVID-19 period. **(B)** Spaghetti plot of individual hemoglobin A1c values, with mean shown as the black line.

During the pre-COVID-19 period, the mean HbA1c was 7.6%, which increased to a mean HbA1c of 8.6% during the COVID-19 period (p=0.0002), (**Table 2**; **Figure 1A**). As shown in **Figure 1** **B**., a linear mixed effect spline model with the knot on March 11^th^, 2020, showed an increase in the slope of HbA1c during the COVID-19 period compared to the pre-COVID period. Compared to the pre-COVID period, where the mean change in HbA1c over time was relatively flat (0.01% per 30-day month), during the COVID period, the mean increase in HbA1c over time was 0.13% per 30-day month (p=0.0397). There was no significant change in the mean or trajectory of weight, BMI, or BMI z-score. These findings held true when adjusted for sex, age, race, ethnicity, duration of T2D diagnosis, insurance type, and virtual school attendance.

**Table 2:** Comparison of unadjusted clinical variables and outcomes pre-COVID-19 compared to during COVID-19.

Variable	Pre-COVID-19 (3/11/2019-3/10/2020)	COVID-19 (3/11/2020-3/10/2021)	Difference (95% CI)	p-value
# Endocrine clinic visits, median (IQR)*	2.0 (2.0)	2.0 (2.0)	0 (NA)	0.2008
# Missed endocrine visits, median (IQR)*	1.0 (3.0)	2.0 (2.0)	1 (NA)	0.4792
# Admissions, median (IQR)*	0 (1)	0 (0)	0 (NA)	0.2277
# Emergency department visits, median (IQR)*	0 (1)	0 (0)	0 (NA)	0.1647
Weight in Kg, mean (SD), n=55**	101.5 (29.4)	102.9 (30.1)	1.41 (-0.65, 3.48)	0.1752
BMI, mean (SD), n=53**	36.0 (9.2)	36.1 (9.4)	0.13 (-0.57, 0.82)	0.7182
BMI Z-score, mean (SD), n=52**	2.1 (0.8)	2.2 (0.7)	-0.04 (-0.11, 0.03)	0.2410
Hemoglobin A1c %, mean (SD) n=57**	7.6 (2.1)	8.6 (2.5)	0.98 (0.49, 1.48)	0.0002

*Wilcoxon signed rank test.

**Paired t-test.

Missing data or weight measurements was due to virtual visits in the COVID-19 period. NA, Not Applicable.

There was no change in the mean number of endocrine visits, missed endocrine visits, ED visits or hospitalizations from the pre-COVID-19 to COVID-19 period. However, in the pre-COVID-19 period, all diabetes clinic visits were in-person, while in the COVID-19 period, 62.8% of visits were *via* tele-medicine. Of note, there were 3 patients who did not have in-person follow up during the COVID-19 period and therefore had missing vitals or laboratory testing. These patients were compared to those that had follow-up data available, and no statistically significant differences were identified in baseline characteristics.

## Discussion

This study showed that youth with known T2D experienced worsening glycemic control during the COVID-19 pandemic. Although a pandemic related increase in obesity has been documented worldwide, we did not see a significant change in BMI, BMI z-score, or weight in this study population. This may be due to a detrimental change in body composition from a lack of physical activity, leading to decreased lean mass with an increase in fat mass. Chronic glucosuria caused by suboptimal glycemic control mitigating weight gain may have also played a role in the lack of significant change in BMI, BMI z-score or weight. Others have theorized that parents working from home resulted in more home-cooked meals and an increase in consumption of fruits and vegetables, which may have also led to reduced weight gain (19). However, data from the United States and internationally suggest that an increase in sedentary behaviors and high calorie snacking in combination with more screen time in response to stress have resulted in weight gain among children and adolescents (19–23). Our findings are similar to a small study from Malaysia of 30 adolescents with T2D who had a significant increase in HbA1c during the COVID-19 pandemic, along with a significant decrease in BMI and weight parameters that they theorized was due to worsened glycemic control and catabolic state (24). However, these results may not be generalizable to our population in the United States. In the United States, T2D disproportionately affects minority and low-income youth (5, 6), which was reflected in this study’s participants of whom 74.6% were African American, and 77.8% had public insurance. Further, this same population has also been significantly impacted by the COVID-19 pandemic (4, 25) and its consequences, including increasing food insecurity (26) and disparities in the success of virtual school (27). The transition from in-person to virtual school led to an increase in sedentary behavior (28), along with an expected increase in screen time and a decrease in physical activity (29, 30). Moreover, the change from in person to video visits (62.8% of endocrine visits during the COVID-19 period) also likely contributed to the worsening of HbA1c during the COVID-19 pandemic. Experience from prior natural disasters, such as hurricane Katrina in Louisiana and the Hull flood in the UK, suggests that management of chronic diseases, including diabetes, is negatively affected even months to years after these catastrophes (31, 32). Indeed, in this study, HbA1c increased significantly in this patient population, a trend that may persist and needs to be addressed by the medical community.

T2D in youth has been shown to be distinct from T2D in adults with poorer outcomes, rapid β cell decline, insulin resistance and weight gain (33). After new diagnosis of T2D many patients are able to discontinue insulin therapy (34); however, there is a high rate of treatment failure requiring therapy intensification with restarting insulin or initiation of other diabetes medication (35). While the American Diabetes Association (ADA) 2021 guidelines recommend intensive lifestyle programs with increased physical activity and healthy eating habits to promote weight loss, adherence is suboptimal. While making recommended and sustainable modifications in diet and physical activity has always been difficult for patients with T2D (36), the COVID-19 pandemic has made implementing positive lifestyle changes even more challenging. Recent data suggests that in youth with T2D, reduction in BMI correlates with improvement in HbA1c, an outcome that is more likely to be achieved with medication other than insulin therapy (37). Chang et al. also found no difference in HbA1c between patients prescribed basal insulin and those prescribed basal plus prandial and correctional insulin, suggesting that providers should consider simplifying insulin regimen. DPP4 inhibitors and SGLT-2 inhibitors, which are being studied in the pediatric population, may expand treatment options, and provide much needed alternatives to insulin’s lipogenic effect. Given the high rate of diabetes associated complications in youth onset T2D, it is important to maintain close patient follow-up and routine monitoring for complications per ADA guidelines (38, 39).

Adolescents with T2D are also at increased risk of depression and anxiety (40). This mental health burden can lead to increased insulin resistance and worsened glycemic control due to less exercise, worse diet, and less medication adherence (41). Some have suggested that children may have benefitted from the increase in time spent with family during the COVID-19 pandemic (42). However, it is likely that fear of COVID-19 exposure to self and loved ones, social isolation, financial strain, and cessation of school-based support systems contributed to worsening of anxiety and depression among youth with T2D (21). This highlights the importance of the ADA recommendation for yearly depression screening, with appropriate referral to mental health resources, which has become increasingly relevant during the COVID-19 pandemic.

This is among the first reports examining the impact of the COVID-19 pandemic on youth with existing type 2 diabetes. While the patient demographics are diverse, this study is limited by the small study sample from a single institution. This study cohort was predominantly minority youth; thus, results may not be generalizable to other populations. The study cohort includes some participants (n=13) who were recently diagnosed with T2D in the year prior to March 10^th^ 2020, which could potentially affect results given they have fewer data points in the pre-COVID period. Also, given the natural progression of T2D they may have improvement in glycemic control shortly after diagnosis, potentially leading to an underestimation of worsening of glycemic control during the study period. As a retrospective study, data regarding school attendance, virtual vs in-person school, parental presence in the home during the COVID-19 period and COVID-19 positivity were not captured consistently in the EMR. Although hospital admission and ED data was collected from the EMR and Care Everywhere, it is possible that participants presented to a hospital system not connected to our EMR and thus ED/hospital admission data may be an underestimate. Because of multiple comparisons made within this study, its findings should be replicated. Larger, prospective, multicenter studies across the United States are recommended to determine the full impact of the COVID-19 pandemic on this high-risk pediatric population with T2D.

In summary, youth with type 2 diabetes experienced a worsening of glycemic control during the COVID-19 pandemic. Providers should work closely with patients to optimize diabetes treatment regimens, while also providing additional resources to address the impact of COVID-19, in an effort to improve glycemic control and prevent long-term diabetes related complications.

## Data availability statement

The original contributions presented in the study are included in the article/supplementary material. Further inquiries can be directed to the corresponding author.

## Ethics statement

This study was approved by the institutional review board at the Johns Hopkins Hospital in adherence to the Declaration of Helsinki, with a waiver of consent.

## Author contributions

RW and SM conceived of the study. TL, AA, SB, MW, KB, extracted the data. EB analyzed the data. SB and RW wrote the manuscript. All the authors have accepted responsibility for the entire content of this submitted manuscript and approved submission.

## Conflict of interest

The authors declare that the research was conducted in the absence of any commercial or financial relationships that could be construed as a potential conflict of interest.

## Publisher’s note

All claims expressed in this article are solely those of the authors and do not necessarily represent those of their affiliated organizations, or those of the publisher, the editors and the reviewers. Any product that may be evaluated in this article, or claim that may be made by its manufacturer, is not guaranteed or endorsed by the publisher.
